# “Phantom akathisia” in an amputated leg of a sarcoma patient: a case report

**DOI:** 10.1186/s13030-020-00178-8

**Published:** 2020-03-07

**Authors:** Mayumi Ishida, Jungo Imanishi, Yasuo Yazawa, Yu Sunakawa, Tomoaki Torigoe, Hideki Onishi

**Affiliations:** 1 Department of Psycho-Oncology, Saitama Medical University International Medical Center, Hidaka, Japan; 2 Department of Orthopedic Oncology and Surgery, Saitama Medical University International Medical Center, Hidaka, Japan; 3 Department of Orthopedic Surgery, Saitama Medical University Hospital, Moroyama, Japan; 4 Department of Clinical Oncology, St. Marianna University School of Medicine, Kawasaki, Japan

**Keywords:** Orthopaedics/sarcoma, Psycho-oncology, Palliative care, Akathisia, Amputation

## Abstract

**Background:**

Akathisia is a rather common extrapyramidal side effect of antipsychotic drugs and antidepressants, often resulting in severe discomfort for patients. However, due to the diversity of symptoms, it is often overlooked. We hereby report a case with akathisia that mainly appeared in an amputated leg.

**Case presentations:**

A 60-year-old woman, who had undergone external hemipelvectomy for a recurrent soft tissue sarcoma, was referred to the Department of Psycho-Oncology due to worsening anxiety and restlessness. She was not unconscious or disoriented. Her chief complains included restlessness, an itching sensation in the area corresponding to the amputated left leg, and a feeling as if the lost left leg were raising itself. Detailed examination revealed that she had been administered 10 mg per day of oral prochlorperazine maleate for nausea induced by the oxycodone that had been prescribed to control post-operative pain. Akathisia was suspected and prochlorperazine maleate treatment was discontinued. All the symptoms were alleviated on the next day, and disappeared in 3 days. Eventually, she was diagnosed with akathisia.

**Conclusions:**

This case indicates that the symptoms associated with akathisia can occur in an amputated extremity. Considering two previous reports of “phantom dyskinesia”, extrapyramidal syndromes may result in unusual presentations if occurring in an amputated extremity. Not only should the use of antipsychotic drugs and antidepressants be carefully considered, but also closer observation of psychological symptoms is required after prescription of these drugs because the clinical presentation of akathisia can be various and confusing due to modifications caused by other factors as in this case.

## Background

Psychological symptoms such as anxiety and depression are related to the quality of life (QOL) of cancer patients. As such, early detection and appropriate evaluation of as well as response to the symptoms associated with these psychologically abnormal conditions are required [1]. It is also expected that the need for improvement in QOL will continue to grow in importance as life expectancy continues to rise, even for advanced cancer patients, thanks to the remarkable recent advances in cancer treatment.

Akathisia is an extrapyramidal side effect of antipsychotic drugs and antidepressants that occurs in nearly 20% of patients receiving such drugs, and it has been reported to be associated with severe discomfort for patients sometimes resulting in suicide [2, 3]. However, due to the diversity of symptoms, it is often overlooked [4]. Our Psycho-Oncology team have reported that akathisia is common among patients who have been diagnosed with cancer and that akathisia can occur even in patients who cannot move due to disease progression [5–8].

Here we report a case of akathisia in a patient who had a lower limb amputated due to local recurrence of a malignant soft tissue tumour. Symptoms indicative of akathisia appeared at the site corresponding to the amputated leg but improved promptly after cessation of treatment with the causative agent.

## Case presentation

**Fig. 1:**
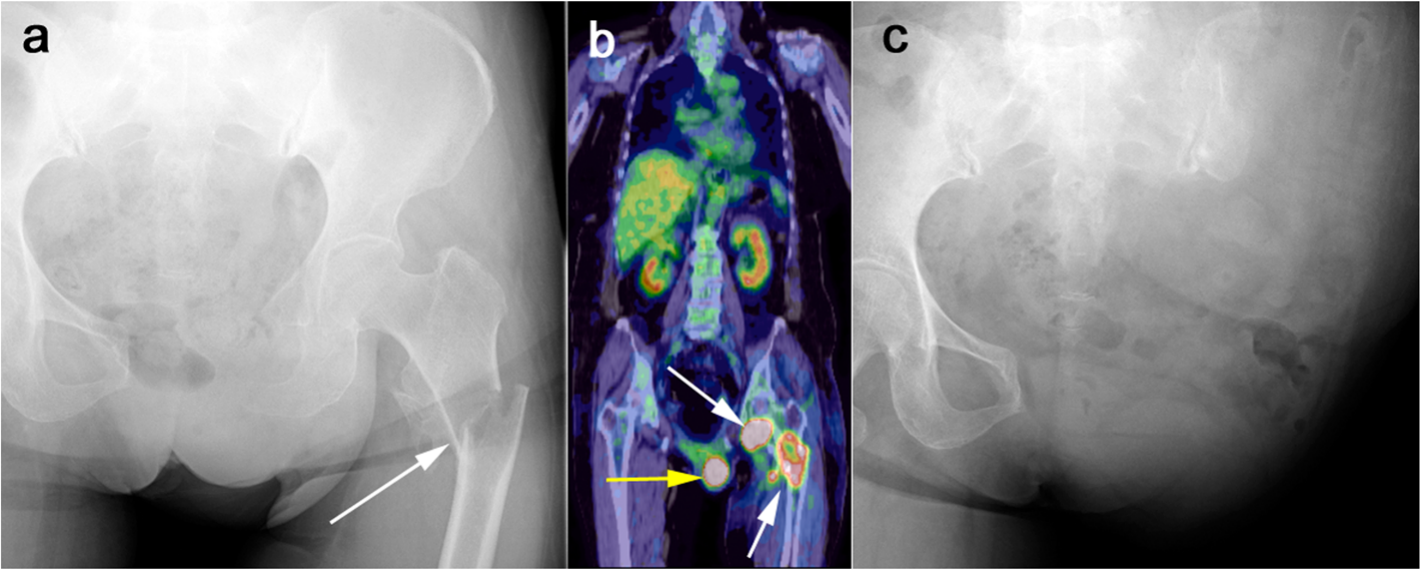
**a** A radiograph showing a pathological fracture of the left proximal femur due to the second local recurrence of extra-skeletal osteosarcoma (a white arrow). **b** FDG-PET scan illustrating two abnormal uptakes (white arrows) indicating local recurrence. The bladder was shifted due to bladder herniation after the resection of the pubic bone for the first local recurrence (a yellow arrow). No other abnormal FDG uptake was detected. **c** Post-operative radiograph showing that the left leg together with the left pelvis had been amputated

**Fig. 2:**
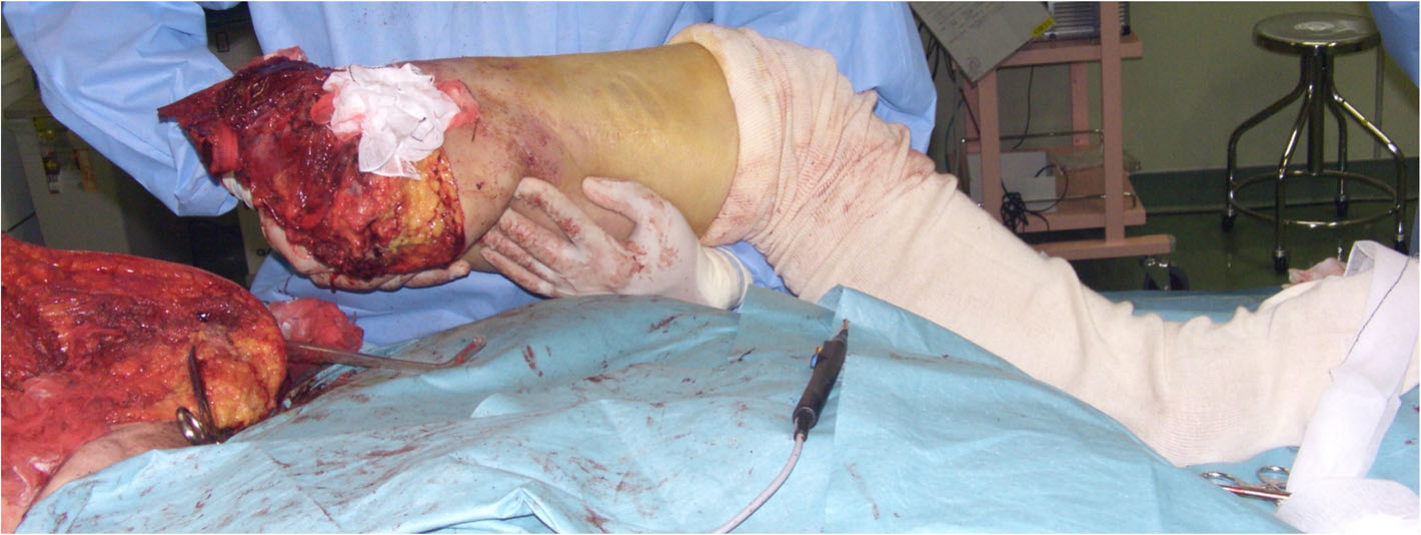
Intra-operative photograph showing that the left leg had been amputated. After the amputation, the bladder was fixed to its original position and covered with a left Gluteus Maximus muscle flap

A 60-year-old woman with advanced soft tissue sarcoma, given oncological treatment in the Department of Orthopaedic Oncology and Surgery and the Department of Clinical-Oncology, was referred to the Department of Psycho-Oncology due to worsening anxiety and restlessness after amputation. She was initially diagnosed with Union for International Cancer Control (UICC) stage IV extra-skeletal osteosarcoma in the left proximal medial thigh, with multiple lung and loco-regional lymph node metastases 4 years previously. After resection of the primary tumour and subsequent adjuvant chemotherapy, these metastases were resolved, and she then underwent careful observation. The first local recurrence occurred 18 months post-operatively, and she subsequently received second-line chemotherapy, followed by resection of the recurrent tumour. Thirty-one months after the first surgery, she developed a second local recurrence with a pathological fracture in the left proximal femur, and underwent external hemi-pelvectomy 1 month prior to referral (Figs. 1 and 2).

After the hemi-pelvectomy, the patient suffered from post-operative pain. On the day after operation (POD 1), continuous intravenous administration of 30 mg/day of oxycodone hydrochloride was commenced. The patient began to experience nausea after the prescription of oxycodone hydrochloride, but no cause other than the opioid was apparent. The dose of oxycodone hydrochloride was reduced gradually, and 10 mg/day of oral prochlorperazine maleate was added from the sixth day post-operatively (POD 6). The nausea disappeared on POD 7, thereafter the wound pain was gradually alleviated and the oxycodone dose was reduced. On POD 32, the patient complained of an anxious feeling and malaise that suddenly appeared, asking “Why am I getting more and more anxious even though one month has already passed since surgery?” These symptoms continued to worsen, and her husband perceived that she was not behaving as she had in the past. She complained that she felt as if her amputated left leg were raising itself, along with an indescribable sense of anxiety and restlessness.

The symptoms continued to worsen and, on POD 34, she said to medical staff “My amputated left leg does not follow my orders, rising on its own.” In addition, she repeatedly shouted “Don’t rise up, please stay down, please! I should not have given my consent to amputation!”. She felt as if her right leg were being tickled inside, and often requested nurses and her husband to rub her right leg, which ameliorated her symptoms. She also expressed worsening restlessness, saying “I cannot stay still in anybody position. Please help me!”

The Psycho-Oncology team reviewed the aforementioned clinical course in detail and interviewed the patient and her husband on the next day (on POD 35); her physical symptoms consisted of the itching sensation in the amputated left leg, the feeling as if the amputated leg were moving itself, and the feeling of restlessness. She was not unconscious or disoriented. Furthermore, prochlorperazine maleate had been administered for 1 month. From the above, her symptoms were considered likely to be akathisia. Administration of prochlorperazine maleate was therefore discontinued.

All the aforementioned symptoms were alleviated the next day (on POD 36), and disappeared completely in 3 days. Phantom pain, which she described as “pricking”, continued until death of disease 14 months after amputation, but the other symptoms never reappeared. The diagnosis of akathisia was eventually confirmed.

## Discussion and conclusions

We reported on an unusual presentation of akathisia in an amputated leg. In this case, after the appearance of anxiety, the patient felt an unpleasant sensation of itchiness in the amputated limb and worsening restlessness with agitation and frustration with her functional impairment. The “Diagnostic and Statistical Manual of mental disorders, 5th edition (DSM-5)” describes medication-induced acute akathisia as “subjective complaints of restlessness, often accompanied by observed excessive movements, developing within a few weeks of starting or raising the dosage of a medication or after reducing the dosage of a medication or after reducing the dosage of a medication used to treat extrapyramidal symptoms” [9]. Except for the impossibility of observing the amputated leg movement, the symptoms in this case report accorded with the aforementioned DSM-5 description, and the symptoms rapidly disappeared after discontinuation of prochlorperazine maleate administration. Therefore, we eventually diagnosed the patient with akathisia. This case indicates that the abnormalities causing akathisia occur in the central nervous system, and the symptoms can occur regardless of the presence or absence of the leg.

The symptoms in this case were characterized by the occurrence of akathisia at the site of the amputated leg. There have been no reports of akathisia in an amputated leg to date, but there have been two reports on “phantom dyskinesia”, both published in the 1980s [10, 11]. Perhaps it would be appropriate to call the abnormal condition in our case “phantom akathisia”, because the condition we witnessed was different from mere phantom pain or mere akathisia. In this case, phantom pain, described as “pricking” by the patient, continued until death of disease, even after all other symptoms had disappeared. Typical phantom pain is described as “shooting”, “pricking”, and “boring” in the past literature [12], whereas the patient in this case report described her strange feeling as “tickling”, which is a common word to describe symptoms of akathisia.

Clinical presentations differ between “phantom akathisia” and mere akathisia, characterised by difficulty in the diagnosis of “phantom akathisia”. In their study of 100 consecutive inpatients starting neuroleptic therapy, Sachdev and Kruk stated that the best clues to discriminate akathisia from non-akathisia were shifting weight from foot to foot or walking on the spot, inability to keep legs still, feelings of inner restlessness, and shifting of body position in the chair [13]. In 2005, Chouinard and Margolese published a manual for rating extrapyramidal symptoms in which they graded the degree of akathisia by the movement of legs and gait [14]. While the importance of evaluating leg movement in the diagnosis of akathisia was highlighted in the two publications, the movement of the “phantom leg” was unable to be observed in our case. Similar phenomena, phantom pain exaggerated or triggered by extrapyramidal symptoms, have been reported as “phantom dyskinesia” [10, 11]. Although the mechanism underlying such exaggeration or triggering of phantom pain by akathisia or dyskinesia is not yet understood, it is certain that diagnosis of extrapyramidal symptoms is difficult and probably prone to under-diagnosis or being overlooked due to its unusual clinical presentation and the invisible involuntary movement in the amputated extremity.

The diversity of akathisia symptoms often results in delayed diagnosis [4], and a delay in diagnosis can cause a severe mental burden on the patient. Indeed, in this case, the patient was overwhelmed mentally, regretting her previous decision to consent to the amputation, but later she regained peace of mind after prompt recovery from akathisia. In general, causative agents of akathisia include antiemetic drugs, such as metoclopramide and prochlorperazine maleate [15, 16], as well as first- and second-generation antipsychotics including haloperidol and chlorpromazine [17]. In a previous study on 20 cancer patients with akathisia, the onset of akathisia after the start of prochlorperazine (*n* = 16), haloperidol (*n* = 3), or chlorpromazine (*n* = 1) ranged from 1 to 285 days, but onset in 70% of the cases was within 1 month, as in our case [7]. It can be said that akathisia typically occurs within 1 month after the start of medication. Medical staff engaged in oncology should consider the possibility of akathisia caused by these drugs, and the medication should be discontinued if symptoms of akathisia are observed.

In conclusion, “Phantom akathisia” can be one of the diverse clinical presentations of akathisia, characterized by anxiety, restlessness, an “itching sensation” in an amputated leg, or a feeling as if the amputated leg were moving by itself. Nausea after chemotherapy, psychotic disorders, and delirium among amputees are likely situations worldwide, and causative agents for akathisia are probably used in many of these cases. By reporting our case, we would like to warn of the possibility of overlooking or under-diagnosing “phantom akathisia” due to its unusual presentation and difficulty in diagnosis.

## Data Availability

Not applicable.
